# Identification of chromatin loops from Hi-C interaction matrices by CTCF–CTCF topology classification

**DOI:** 10.1093/nargab/lqac021

**Published:** 2022-03-08

**Authors:** Silvia Galan, François Serra, Marc A Marti-Renom

**Affiliations:** CNAG-CRG, Centre for Genomic Regulation (CRG), Barcelona Institute of Science and Technology (BIST), Baldiri i Reixac 4, 08028 Barcelona, Spain; CNAG-CRG, Centre for Genomic Regulation (CRG), Barcelona Institute of Science and Technology (BIST), Baldiri i Reixac 4, 08028 Barcelona, Spain; CNAG-CRG, Centre for Genomic Regulation (CRG), Barcelona Institute of Science and Technology (BIST), Baldiri i Reixac 4, 08028 Barcelona, Spain; Centre for Genomic Regulation (CRG), Barcelona Institute of Science and Technology (BIST), Dr. Aiguader 88, 08003 Barcelona, Spain; Universitat Pompeu Fabra (UPF) 08002 Barcelona, Spain; ICREA, Pg. Lluís Companys 23 08010 Barcelona, Spain

## Abstract

Genome-wide profiling of long-range interactions has revealed that the CCCTC-Binding factor (CTCF) often anchors chromatin loops and is enriched at boundaries of the so-called Topologically Associating Domains, which suggests that CTCF is essential in the 3D organization of chromatin. However, the systematic topological classification of pairwise CTCF–CTCF interactions has not been yet explored. Here, we developed a computational pipeline able to classify all CTCF–CTCF pairs according to their chromatin interactions from Hi-C experiments. The interaction profiles of all CTCF–CTCF pairs were further structurally clustered using self-organizing feature maps and their functionality characterized by their epigenetic states. The resulting clusters were then input to a convolutional neural network aiming at the *de novo* detecting chromatin loops from Hi-C interaction matrices. Our new method, called LOOPbit, is able to automatically detect significant interactions with a higher proportion of enhancer-promoter loops compared to other callers. Our highly specific loop caller adds a new layer of detail to the link between chromatin structure and function.

## INTRODUCTION

The human genome consists of 2 m of DNA and its folding in the cell nucleus is not random (1). During the last decade, the three-dimensional (3D) organization of the genome has been associated with the regulation of multiple nuclear functions like DNA replication, repair, rearrangement and recombination, RNA processing and transcription (1–4). From chromosome territories (5) to chromatin compartments (6), topologically associating domains (TADs) (7,8) and finally loops (9), the hierarchical organization of the genome has been gaining in details with the outcome and further improvement of the high-throughput Chromosome Conformation Capture (3C) technologies (6). This detailed characterization of the chromatin structure also helped us to characterize the roles of specific proteins, with perhaps being the CCCTC-binding factor (CTCF) protein as the most striking example.

CTCF is a transcription factor formed by 11 DNA-binding Zinc finger (ZF) found enriched in TAD borders and facilitates mammalian enhancer-promoter loops (10). Its functionality is dependent on the location and the relative orientation of its binding sites. Interacting CTCF pairs tend to be organized in a convergent orientation, with the binding motifs facing each other (9). The distribution and organization of CTCF leading to TAD formation has been explained by the loop extrusion model that involves CTCF but also other protein complexes like cohesin (11,12). Briefly, the cohesin complex, which forms a ring-shaped structure upon loading onto chromatin, extrudes chromatin resulting in a growing loop of DNA. The extrusion is blocked on both sides when cohesin encounters two CTCF in a convergent orientation. Various experiments have described the relevance of the binding motif orientation for loop formation by inverting or deleting CTCF binding sites using CRISPR/Cas9 experiments (12,13). Moreover, a number of studies revealed the effect of CTCF depletion, resulting in a fainting of TADs and loop structures, while maintaining compartment organization. It suggests that CTCF and cohesin play a role in TAD and loop formation, but not necessarily in genome compartmentalization (14,15).

Recently, several studies have applied Aggregate Peak Analysis (APA) (9), or pile-up methods (16) to analyze Hi-C datasets using mean signals from selected regions of interest (17–22), such as CTCF loops. At the level of single chromatin loops, their biological relevance in gene regulation, has led to the development of several loop callers. However, most of the methods present low reproducibility between biological replicates and a low correlation with different biological markers (23). Here, we propose to use self-organizing feature maps (SOFM) (24), an artificial neural network, to classify the signal from pairs of CTCF without any prior information about their topological organization. This approach allows us to obtain sub-populations of CTCF–CTCF interacting structures and to identify their epigenetic signature in an unsupervised manner. We then hypothesize if this link between structure and epigenetic signature could be used to identify regions with specific activity from their structural features only. As a proof of concept, we developed LOOPbit, a loop caller that takes as input a Hi-C interaction matrix and predicts the localization of loops. LOOPbit is based on a convolutional neural network (CNN) model trained on the loops defined in selected SOFM clusters with an epigenetic signature corresponding to active chromatin. An input Hi-C interaction matrix is then scanned with the CNN model, and LOOPbit outputs the probability that a given region matches the CNN model. Although CNN model can be trained on any Hi-C interaction matrix, provided CTCF binding data, in this work we used only the model generated in GM12878. We show that LOOPbit is able to identify chromatin loops at similar levels as other loop callers, but with enrichment in enhancer-promoter contacts.

## MATERIALS AND METHODS

### ChIP-seq and Hi-C data

CTCF ChIP-seq experiments of Human B-lymphocyte cell line (GM12878) were downloaded from the ENCODE database (https://www.encodeproject.org/; dataset ids: ENCSR000DRZ, ENCSR000AKB and ENCSR000DZN) (25). After processing the peaks following the ENCODE pipelines available at https://github.com/ENCODE-DCC, only common peaks from all three experiments were kept resulting in a total of 52 844 CTCF peaks. Next, the orientation of the CTCF binding motifs was assessed by means of the MEME and FIMO motif-based sequence analysis tools (26,27). Only peaks with a statistically significant CTCF motif were kept (*P*-value < 0.05), which resulted in a total of 41 816 *ChIP-seq + motif* peaks. This filter discarded ∼21% of the original detected CTCF ChIP-seq peaks that were missing the canonical CTCF binding motif in the predicted peak region. Additionally, we downloaded ChIP-seq experiments against RAD21 and SMC3 for GM12878 from the encode project (dataset ids: ENCSR000BMY and ENCSR000DZP for RAD21 and SMC3, respectively). Peaks for those datasets where processed following ENCODE pipeline with no further filtering.

*In situ* Hi-C datasets for GM12878 cell line (9) were downloaded from the GEO database (Supplementary Table S1) and its replicates merged and parsed using the TADbit as previously described (28). Two resolution matrices (i.e. at 100 kb and at 5 kb) were obtained and normalized using OneD (29) with default parameters. The 100 kb Hi-C matrices were next used to calculate chromosome compartmentalization (6,30) using TADbit (28).

To generate the submatrices, we followed the steps of an APA (9) without aggregating the peaks. The 5 kb resolution matrices were further parsed to subtract 45 kb squared submatrices. Each submatrix was centered in both axes on a pair of the 15 597 isolated CTCF *ChIP-seq + motif* peaks (i.e. ∼38% of all selected peaks above). Isolated, or non-overlapping, peaks were defined as those *ChIP-seq + motif* peaks with no other peak within a 50 kb window span from the center of the peak. This additional filter ensured that the observed signal in a Hi-C submatrix was due to a particular pair of CTCF–CTCF peaks and not multiple pairs. Finally, we subtracted a total of 130 655 submatrices between any pair of isolated CTCF–CTCF peaks spanning any distance between 45 kb and 1.5 Mb, which ensured to select most pairs within the size of a typical TAD in the human genome (∼900 kb). These steps leading to aggregate CTCF peaks were performed using *Meta-Waffle* and available in GitHub (https://github.com/3DGenomes/metawaffle).

### Submatrix analysis, deconvolution, classification and clustering

Next, and also using Meta-Waffle, we analyzed, deconvolved and classified the submatrices into micro-clusters, or neurons, using a Self-Organizing Feature Map (SOFM) approach (24) available in http://neupy.com. Briefly, and for this application, *Meta-Waffle* extracted the previously mentioned 45 kb submatrices (9 × 9 submatrices at 5 kb resolution) from an input full Hi-C interaction matrix. The 130 655 submatrices were next sigmoid transformed to scale their values between 0 and 1. These re-scaled submatrices were then input to the SOFM and classified in clusters or, following the SOFM terminology, *neurons*. To obtain the most pertinent SOFM clustering, we tested several combinations of parameters (examples of output in Supplementary Figure S1). We tried: 10 × 10, 20 × 20, 30 × 30, 40 × 40, 45 × 45 and 50 × 50), learning radius (i.e. 1, 2 and 5), standard deviation (i.e. 0.5, 0.1 and 0.01), steps (i.e. 1, 0.5, 0.1 and 0.01) and Epochs (i.e. 30, 90, 130 and 200). Combination of the tested parameters resulted in a total of 1152 generated SOFMs. Assessing which set of parameters results in the best submatrices classification is not trivial as there is no gold standard for the classification of CTCF–CTCF interaction submatrices. Therefore, we devised three evaluation metrics to select the SOFM optimal parameters. First, a percentage of classified submatrices in order to minimize the number of singletons SOFM neurons (with only one CTCF–CTCF submatrix). Second, the variability between neurons (the more distinct the better). And third, a compartment segregation score to maximize the segregation of the two main genome compartments within the SOFM grid. Compartments were measured using the first eigenvector of a transformation of a chromosome Hi-C interaction matrix (6). Each value in this eigenvector represented the belonging to a compartment type of a given genomic bin. We used positive values for A compartments and negative values for B compartments. We defined the compartment segregation score as the absolute of the average eigenvector value of all genomic regions in a given neuron. The SOFM optimal parameters, that maximized all the three evaluation metrics proposed, were: grid size: 30 × 30; learning radius: 5; standard deviation: 0.5; step: 0.01 and epochs: 30.

With the dataset of submatrices projected onto the 2D SOFM map, we used the coordinates of each SOFM neuron to infer groups of CTCF–CTCF interaction patterns. At this stage SOFM neurons represent groups of topologically similar pairs of CTCF–CTCF ChIP-seq peaks; each neuron can be represented as an average structure by averaging all submatrices it contains. From the generated 2D SOFM map, we next clustered the neurons using their average submatrix (a representation of the 3D structure of the chromatin observed between pairs of CTCF ChIP-seq peaks). To perform the clustering, we first generated a 2D embedding using the Uniform Manifold Approximation and Projection (UMAP) (31). In contrast with other embedding methodologies like t-SNE (32), UMAP can be used as an effective pre-processing step to enhance the performance of density-based clustering. The UMAP was computed using a local neighboring size of 6 for the manifold approximation, an effective minimum distance between embedded points of 0.3 and a number of epochs of 100 to increase its accuracy. The final 10 clusters of Hi-C CTCF–CTCF neurons were obtained with HDBSCAN (33) with default parameters, considering a minimum of six for the cluster size and a minimum of six neighbors for a point to be considered a core point.

### Chromatin states integration

The chromatin 15-states model for the GM12878 cell line was downloaded from the Roadmap Epigenomics Consortium (34). Following a similar previously published protocol (23), all 15 states were merged into four major classes: promoter (Active TSS, Flanking Active TSS, Bivalent/poised TSS Flanking bivalent TSS), enhancer (enhancers, genic enhancers, bivalent enhancers), repressed polycomb (repressed polycomb, weak repressed polycomb) and heterochromatin (heterochromatin, quiescent/low). Next, and also following the methodology in (23), the genome was segmented into 5 kb bins, and each bin was classified based on the overlap (>50 bp) with any of the four major chromatin states. A bin could be assigned to one or more categories. Finally, interactions between bins were classified as ‘not expected’ (interacting promoter/enhancer bin with heterochromatin bin), ‘promoter–enhancer’ (interacting promoter bin with an enhancer bin) and ‘heterochromatin–heterochromatin’ (interacting heterochromatin bins). Note that submatrix annotation is not exclusive and the same CTCF pair can be classified into more than one category (e.g. a ‘promoter–enhancer’ submatrix can also be ‘heterochromatin–heterochromatin’).

### The LOOPbit CNN

LOOPbit is a Convolutional Neural Network (CNN) trained to predict the localization of loops from Hi-C interaction matrices. LOOPbit was trained using the TensorFlow platform (https://www.tensorflow.org) with the following standard workflow: input matrix flattening—dense layer of 1024 neurons (with ReLu activation function)—dropout (set to 0.2 to overcome overfitting)—dense layer of 4 neurons (with Softmax activation function). As an input, the CNN takes tensors of shape (9, 9) that will be flattened in the first layer. The dense layers were used to perform the classification of input Hi-C submatrices into two different classes: loop and no-loop. In order to avoid potential genomic context overfitting (35) the dropout ratio was set to 0.2 (36) even-though the total number of free parameters (88,064) is relatively low. Higher dropout ratios may be set for more complex designs. The LOOPbit CNN was trained by a 20% leave-out of the data used as test and 80% of the data for training. The training dataset obtained by sub-sampling a total of 6,000 randomly selected CTCF–CTCF submatrices from the SOFM neurons with clear signal of looping (loops) and another 6000 randomly selected CTCF–CTCF submatrices from the SOFM neurons with no signal of looping (no-loop). In particular, loop submatrices were extracted from the HDBSCAN clusters 1, 2, 3, 4 and 5, and no-loop submatrices from HDBSCAN clusters 7, 8, 9 and 10 (Results). The trained model resulted in a classification accuracy of 85.9% and 89.6% for the loop and no-loop class, respectively.

### LOOPbit benchmark

The CNN model trained on the cell line GM12878, was then used to predict loop localization in the benchmark dataset proposed by Forcato *et al.* (23) that consists of 36 previously published Hi-C datasets from different cell lines and organisms (Supplementary Table S2). The Forcato's benchmark dataset was selected as it represents the most exhaustive dataset proposed to date to benchmark loop-caller accuracy. Moreover, Forcato's benchmark provides the results of previously benchmarked six loop-callers and compiles a set of metrics measured at different resolutions. To avoid biases derived from the data processing pipelines, all 36 datasets were processed using the same protocol as the training datasets from GM12878 cell line (see1 above). Then, the Hi-C experiments were analyzed with LOOPbit, scanning chromosome-wise using a window of 9 × 9 bins and a step of 1 bin, to predict loops at two different resolutions (5 kb and 40 kb). The 5 and 40 kb resolutions were chosen to match the previously executed benchmarks using exactly their same accuracy metrics on our set of predicted loops (23). First, the Jaccard Index to assess reproducibility between replicates of the same experiment. Two predicted loops were considered to be identical when they shared exactly the same anchoring bins in both replicates. Second, to characterize the possible biological relevance of the predictions, the enrichment of diverse chromatin marks at loop anchors was calculated. For the benchmarking, the 15-states chromatin models for GM12878, IMR90 and h1-ESC cell lines were downloaded from the Roadmap Epigenomics Consortium (34) and analyzed as previously described (23). For the fly late embryos dataset, the 16-chromatin states model was downloaded from modENCODE (37). As previously, the states were also merged into four major classes: promoter (promoter), enhancer (enhancer 1, enhancer 2), repressed polycomb (PC repressed 1, PC repressed 2), and heterochromatin/low (heterochromatin1, heterochromatin 2, low signal 1, low signal 2, low signal 3). Similarly, the enrichment analysis of the chromatin states at the loop anchors was done as described above. Additionally, the presence of CTCF binding sites and their orientation in predicted loops was assessed only for *cis* interactions identified in the Hi-C maps at 5 kb resolution. Briefly, CTCF ChIP-seq experiments were downloaded (Supplementary Table S3) and peaks processed using *HOMER Motif Analysis* with default parameters. The *cis* interactions conserved in at least two replicates within each dataset (with exception of Jin H1-hESC with one replicate) were intersected with the list of motifs of the CTCF peaks. Then, an interaction was considered convergent if the upstream interacting bin contained one CTCF motif on the forward strand (+ orientation), and the downstream interacting bin contained one CTCF motif on the reverse strand (− orientation) (23).

## RESULTS

### Classifying CTCF–CTCF interactions using Self-Organizing Feature Maps (SOFMs) and Uniform Manifold Approximation and Projection (UMAP)

**Figure 1.**
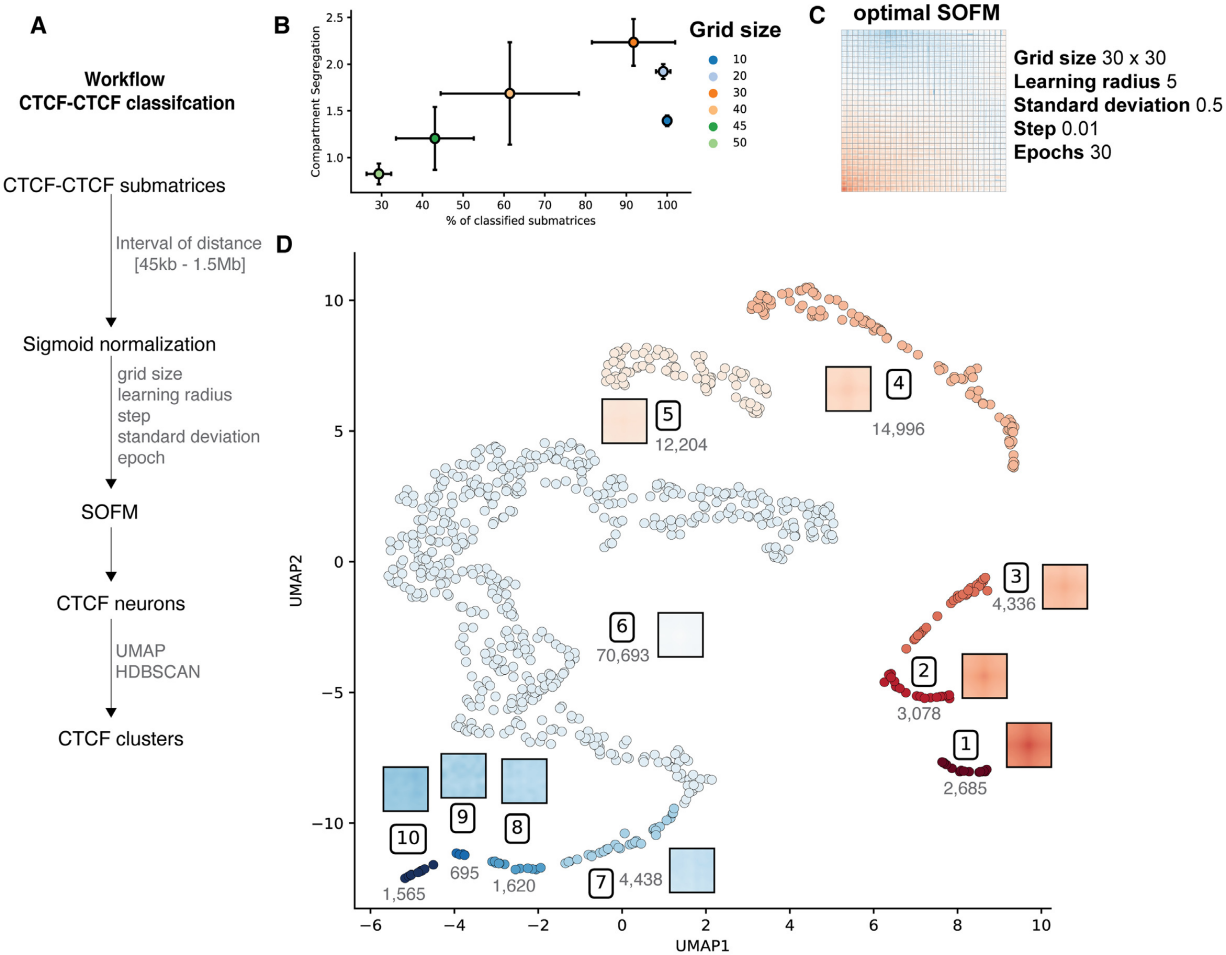
General overview of signal structure deconvolution. (**A**) Schematic workflow to obtain CTCF clusters. (**B**) SOFM parameters selected based on the percentage of classified matrices and the compartment segregation value (see also Supplementary Figure S2). (**C**) SOFM map showcasing the medoid of each neuron for optimal SOFM with the highest compartment segregation as well as highest percentage of classified CTCF–CTCF submatrices. (**D**) Low dimensional representation of the SOFM neurons using a UMAP algorithm followed by a clustering method using HDBSCAN. A total of 10 clusters were obtained. Each point corresponds to a neuron in the SOFM map in panel C. Clusters are represented by the medoid signal and the number of CTCF–CTCF submatrices per cluster.

SOFMs are dependent on several parameters, such as the grid size (number of neurons or clusters), the learning radius, the step size, the standard deviation and the number of iterations or epochs (24). To unveil the best combination of SOFM parameters in the context of CTCF–CTCF submatrices classification, we first extracted the Hi-C submatrices of all possible *cis* pairs of CTCF peaks linearly separated by between 45 kb and 1.5 Mb (Figure 1A; Supplementary File 1). These submatrices, here referred to as ‘CTCF–CTCF submatrices’, spanned over 45 kb in both axes. Each axis representing 20 kb upstream and 20 kb downstream with the central 5 kb bin containing a CTCF peak. Next, all extracted CTCF–CTCF submatrices were input to a SOFM. In total we generated 1152 SOFMs, each with different combinations of parameters (Materials and Methods). To select the optimal parameters, we assessed three measures: (i) the percentage of classified submatrices, considering singletons (SOFM neurons with only one submatrix) as non-classified; (ii) variability between neurons and (iii) average compartment-type segregation across neurons. These three quality measures aimed at identifying the ‘best’ classification that maximized the number of CTCF–CTCF submatrices classified, the separation between neurons (i.e. increased variability inter-neuron), and the homogeneous compartment type within each neuron. Of all varied parameters, the SOFM grid size and the SOFM step size were the most sensitive to the final classification (Figure 1B, Supplementary Figures S1 and S2). The three measures mentioned above were optimal for grid size of 30, learning radius of 5, standard deviation of 0.5, step of 0.01 and number of epochs of 30. Such optimal SOFM map was represented as a grid map where each cell (neuron) is composed by a set of similar CTCF–CTCF submatrices represented by its medoid submatrix (Figure 1C). The SOFM map clearly reflects a variety of CTCF–CTCF submatrices from loop forming pairs (lower-left corner) to non-interacting pairs (upper-right corner). Next, the resulting 900 SOFM neurons were further classified by computing the Euclidean distance between the medoid submatrices of each pair of SOFM neurons and projecting it into a two-dimensional UMAP, a non-linear manifold dimension reduction (31). We finally applied, on the UMAP coordinates of each SOFM neuron, a density-based clustering algorithm (HDBSCAN) (33). This methodology resulted in 10 clusters of SOFM neurons, each representing unique CTCF–CTCF pairing patterns. Clusters go from the most structured with the canonical cross pattern (cluster 1 including 11 SOFM neurons with a total of 2685 CTCF–CTCF submatrices) to a completely flat pattern with no interaction (cluster 10 with 7 SOFM neurons and 1565 CTCF–CTCF submatrices) (Figure 1D). Our results suggest that, beyond the expected contact/no-contact between CTCF–CTCF pairs, we observe a variety of intermediate, well-defined, topological signatures. Next, we set ourselves to functionally characterize each of the detected signatures.

### Functional characterization of the CTCF–CTCF clusters

**Figure 2.**
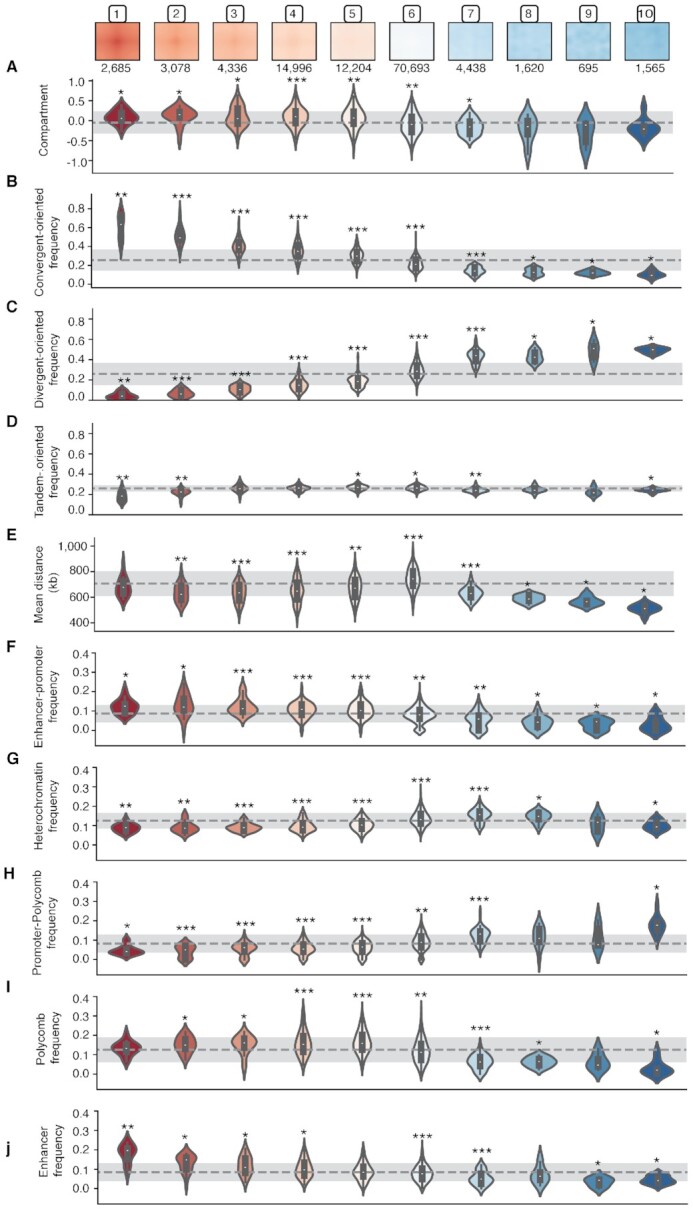
Distribution of multiple genomic features throughout CTCF clusters. In the first row the representative medoid of each CTCF cluster is represented. Below the different genomic features per cluster. In the first row the compartment type distribution, then the binding CTCF motif orientation, the distance between the two CTCFs and finally the chromatin state enrichment of the CTCF pairs. The dashed gray lines mark the mean, whereas the standard deviation is marked with lighter gray. Statistical significance against the mean is calculated using Wilcoxon test. *P* < 0.05 (*), *P* < 0.001 (**), *P* < 0.0001 (***).

As described before, segregation of A/B compartment types between neurons was used as a metric to select the optimal set of parameters for the SOFM classifier. However, this measure was agnostic to the medoid submatrix topology used to cluster neurons. Our results confirm that clusters with clear loop topology (clusters 1 to 5) are enriched in A-type compartments at the anchor points of the loops. Conversely, less structured clusters, with non-interacting CTCF–CTCF (clusters 6–10), are enriched in B-type compartments (Figure 2A). The separation between clusters 1–5 and 6–10 is also observed when revealing enrichment in CTCF pairs with different directionality (9), CTCF–CTCF clusters with clear interaction patterns are enriched in convergent-oriented CTCFs (clusters 1–4, Figure 2B), while non-interacting patterns are enriched in divergent-oriented CTCFs (clusters 7 to 10, Figure 2c), and parallel-oriented CTCFs are slightly enriched in mid-interacting signal clusters (clusters 5 and 6, Figure 2D). Note that in these CTCF–CTCF orientation categories, significant enrichment in a category is usually accompanied by significant depletion in the others. Next, we observed that CTCF–CTCF clusters with clear loop topology (clusters 1–3) have a mean genomic distance between CTCF peaks of between 634 and 680 kb (Figure 2E), while clusters 7–10, more enriched in B compartment and divergent orientation, present shorter mean genomic distances spanning from ∼510 to ∼629 kb (Figure 2E). Note that this measure may be affected by our strict pre-selection of non-overlapping CTCF sites (Materials and Methods) resulting in a relatively sparse dataset, and, on average, more separated anchors.

To assess whether chromatin states correlate with the CTCF–CTCF clusters, we next measured chromatin state enrichment at CTCF sites for each cluster (Materials and Methods). Interestingly, pairs of CTCFs that form loop-like structures (clusters 1–4) are enriched with enhancer-promoter interactions (with one anchor point labeled as ‘enhancer’ and the other as ‘promoter’, Materials and Methods) (Figure 2F). Anchors falling in the ‘heterochromatin’ state have an almost opposite distribution: enriched in clusters 6–8 (Figure 2G). Interestingly, CTCF–CTCF cluster 10, which corresponds to the most B compartment cluster, present less heterochromatic anchors than expected but, in turn, is enriched in polycomb-promoter pairs (Figure 2H). CTCF–CTCF pairs with polycomb in both anchors are strongly enriched in mid-interacting clusters (clusters 4–6) and depleted in non-interacting pairs (clusters 7–10) (Figure 2I). Finally, enhancer-enhancer pairs, are more present in interacting clusters (clusters 1–4) and less abundant in non-interacting clusters (clusters 7–10) (Figure 2J). Interestingly cluster 1, with the strongest interaction pattern, presented the highest concentration of enhancer-enhancer loops, being this feature, it's most distinct measure when compared to cluster 2 or 3. Finally, and in agreement with previous results, we noticed an enrichment for cohesin subunits (RAD21 and SMC3) in most active clusters (Supplementary Figure S3).

Our analysis indicates the existence of clusters or types of CTCF–CTCF pairs that gradually expand from enhancer-promoter, convergent, mid-range interacting pairs in A-type compartment, to a polycomb-heterochromatin, divergent, short-range, non-interacting pairs in B compartment. Taken together, our results based solely on the interaction pattern of CTCF–CTCF proteins highlights two expected major types of CTCF–CTCF pairing, one more active with clear interactions, and one more heterochromatic without interactions. Moreover, the well-defined clusters with intermediate levels of activity do not correspond to the expected intermediate functional profiles as exemplified by the cluster enriched in polycomb states.

Based on these results, we hypothesized that SOFM clusters could be used to train a convolutional neural network (CNN) and generate a highly specific model to detect CTCF-bound promoter-enhancer loops. Moreover, if validated, this hypothesis would help characterize the link between chromatin loop structure and function.

### Loop calling using LOOPbit, a CNN trained with looping and non-looping CTCF–CTCF pairs

**Figure 3.**
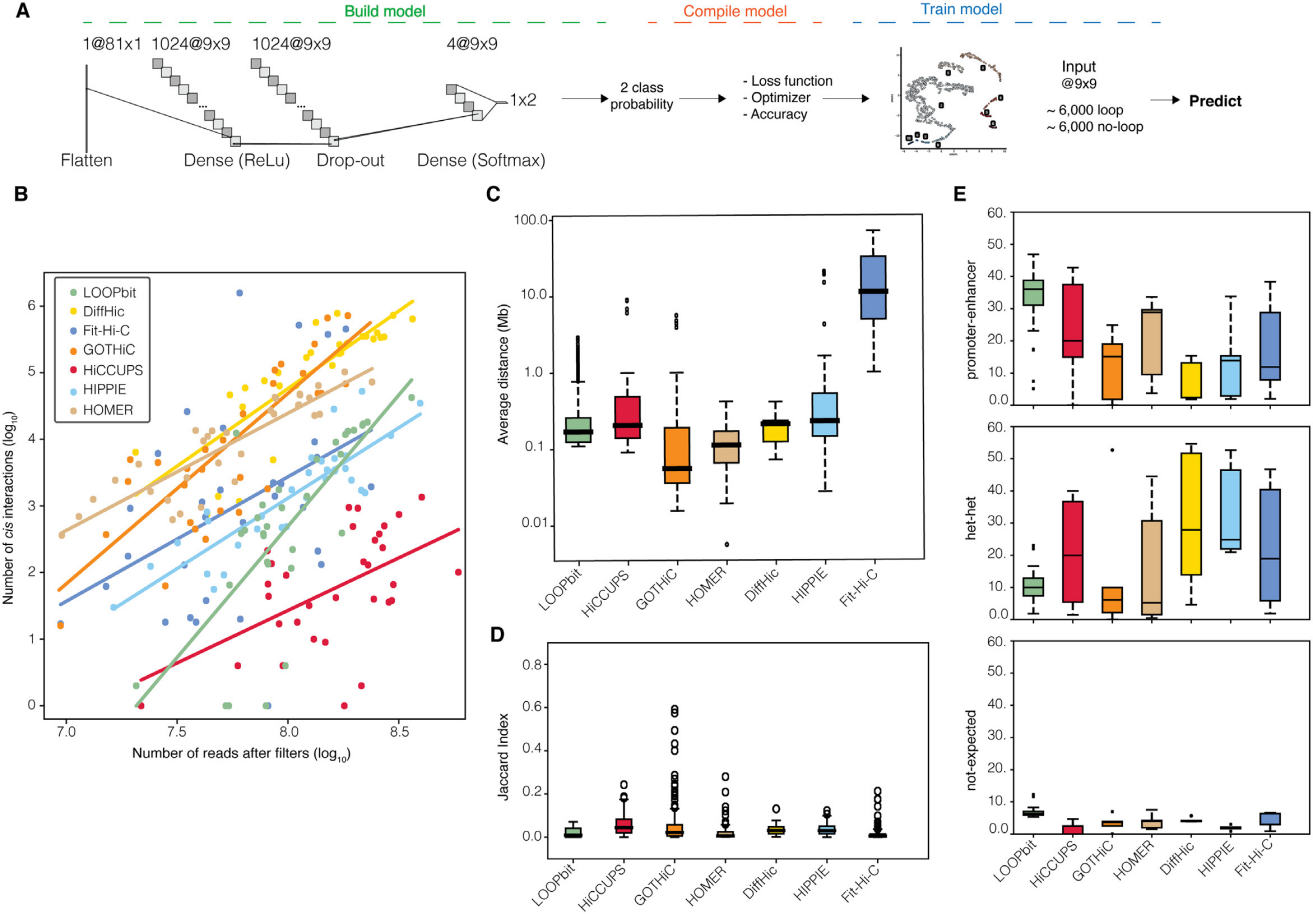
LOOPbit technical and biological benchmarking. (**A**) CNN workflow, model building with the multiple layers to transform the input data. Model compilation to assess the accuracy and optimize the model. Finally, model training using the data coming from the CTCF–CTCF SOFM deconvolution. (**B**) Representation of the number of reads after filters and the number of identified cis-interactions by LOOPbit in all experiments at a 5 kb resolution (n = 32). (**C**) Average distance between the identified loop-anchors of all the Hi-C experiments at 5 kb resolution (*n* = 32). (**D**) Boxplot representing the Jaccard Index, in here the overlapping between exact loop-anchors were considered to be the same loop between the same replicates (*n* = 39). (**E**) Proportion of the identified cis interactions based on the chromatin states at their anchoring points of the datasets at 5 kb resolution (*n* = 32).

Next, we randomly subset a total of 6000 CTCF–CTCF submatrices from clusters 1 to 5 as loop forming pairs (significantly enriched in CTCF-bound promoter-enhancer loops) and another 6000 CTCF–CTCF submatrices from clusters 7 to 10 as non-loop forming pairs. The two datasets, loop and non-loop, where next used to train a CNN that we called LOOPbit. The CNN aims to automatically assign a probability of being a loop to any 9 × 9 submatrix in a genome-wide Hi-C interaction matrix (Figure 3A and Materials and Methods). LOOPbit, which was trained by a 20% leave-out of the used data, was then assessed for accuracy and compared to other loop-calling methods using a recently published benchmark (23). LOOPbit, similarly to other published methods (including HiCCUPS (9), GOTHiC (38), HOMER (39), diffHic (40), HIPPIE (41) and Fit-Hi-C (42)), detects an increasing number of chromatin loops in denser Hi-C experiments (Figure 3B). A solution to minimize this effect is to reduce data resolution (i.e. from 5 to 40 kb) (Supplementary Figure S4a). We note here that LOOPbit is the loop caller with steeper slope, meaning that it is the most conservative in sparse dataset. Loops detected by LOOPbit were of similar average size as of HiCCUPS, diffHiC and HIPPIE (∼200 kb) and larger than those by GOTHiC (∼80 kb) or HOMER (∼100 kb) but shorter than those by Fit-Hi-C (∼10 Mb) (Figure 3C). This loop size measure is again dependent on the resolution of the data as loops called at 40 kb resolution increased to ∼1 Mb of size for all methods (Supplementary Figure S4b).

**Figure 4.**
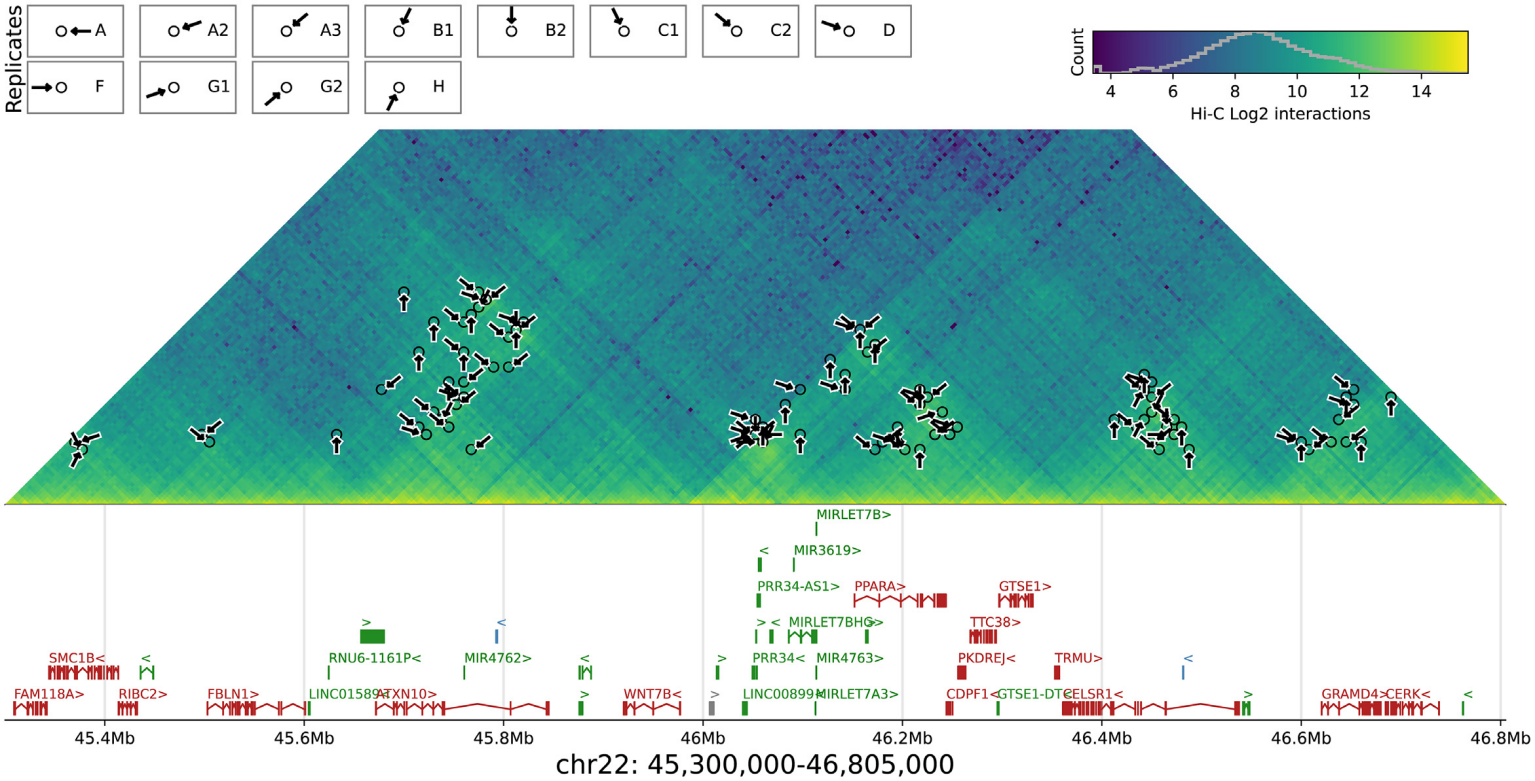
Example of LOOPbit results in several GM12878 replicates (5 kb resolution). Result of loop calling in a chunk of human chromosome 22. Arrows indicate predicted loops in different sub-samples of GM12878 (different arrow orientations correspond to different sub-samples). The Hi-C matrix represents the sum of interactions from all GM12878 replicates available.

Next, we assessed the ability of LOOPbit to reproduce loop detection by measuring the Jaccard Index using replicates of previously published Hi-C experiments (Materials and Methods and Supplementary Table S2). Despite that LOOPbit yields continuous probability clouds instead of pinpointing single cells in the matrix, we used the exact same benchmark measures previously published (23) to calculate the Jaccard Index between replicates (i.e. to consider two loops identical if both their anchoring bins are the same). With such benchmark, LOOPbit results are slightly more reproducible in average than compared to HOMER and Fit-Hi-C, similar to GOTHiC, diffHiC and HIPPIE but lower than HiCCUPS (Figure 3D). We also noticed for LOOPbit an increase in its reproducibility (in terms of Jaccard Index) in denser HI-C datasets. This result is expected as denser matrices contain more information but also because LOOPbit was trained in a dense Hi-C interaction matrix. Importantly, the Jaccard Index, as defined by Forcato *et al.* (23) is very strict. Allowing a looser overlap between loops increased dramatically the measured reproducibility (Supplementary Figure S5), an example of the overlap between sub-samples in GM12878 can be seen in Figure 4 and in Supplementary Figure S6. Finally, we compared the accuracy of the loop callers in terms of the biological relevance of the predicted loops. LOOPbit was able to detect higher percentage of enhancer-promoter loops than any other caller (Figure 3E, top panel). Concomitantly, LOOPbit also detected less loops between heterochromatic anchors (Figure 3E, middle panel) and has similar levels of non-expected loops (that is, between enhancer and heterochromatin, Figure 3e, bottom panel). This result was confirmed by segregating loops by A/B-compartment types, which indicated that heterochromatic loops were enriched in B-compartments while promoter-enhancer loops were enriched in A-compartments (Supplementary Figure S7). Interestingly, and likely particular to LOOPbit as it was trained using CTCF–CTCF selected loops (Materials and Methods), ∼56% of all detected loops in the training had an annotated CTCF site in both anchor points. To note that computationally, LOOPbit had higher requirements than some of the benchmarked methods, taking about 24 h to complete the scanning of a 100 Mb length Hi-C map using a single CPU, and requiring 32 Gb of RAM for the task.

Altogether our results indicate that, although LOOPbit has a similar level of reproducibility as other loop callers, chromatin loops detected by LOOPbit show a clear enrichment in functional signatures when compared to the other methods.

## DISCUSSION AND CONCLUSION

In this work, we introduce the use of the structure signal deconvolution in the context of colocalizing DNA-binding proteins. This methodology aims at identifying clusters of different structural patterns. Until now, most methods detecting structural patterns associated to DNA-binding proteins use aggregate peak analysis (APA) to show an average interaction pattern for different targets of interest. Unfortunately, APA is blind to small subsets with specific interaction patterns, subsets with different structures that could potentially be related to specific functions.

Here, we convoluted the genomic average CTCF–CTCF interaction pattern, and, based solely on this structural feature, were able to characterize distinct subpopulations. According to their structural pattern of interaction only, ten CTCF clusters were obtained (Figure 1D). Each CTCF–CTCF cluster has a specific genome compartment location and epigenetic state. The first observation is that the genomic distance between the CTCF pairs as well as their orientation are relevant features to classify CTCF–CTCF interactions between those that structurally form and do not form loops (1,9). Also expected, loop forming clusters (that is, those from cluster 1 to cluster 5) are enriched in A compartment and drive primarily enhancer-enhancer and enhancer-promoter interactions. In this category, we noted that the first cluster, presenting the strongest pattern of interaction, and the largest genomic distances, was particularly enriched in enhancer-enhancer loops, suggesting transcriptional hubs with rosette like structures (43). On the other side of the spectrum, clusters of CTCF–CTCF pairs presenting sparse or blur signal of interactions are enriched in B-compartment type, heterochromatin or polycomb chromatin states and are spanning shorter genomic distance. These clusters are representative of either silent chromatin (44) or of polycomb-polycomb driven interactions (45,46). Interestingly, we could capture the polycomb interacting network, which has been observed to be essential for cell differentiation and identity. Thus, cluster number 10, which is mostly associated to compartment B, no loop structure, few interactions, divergent CTCF binding sites and short genomic distances between anchor points, contained a surprising enrichment of promoter-polycomb states which could be explained by polycomb protecting a given promoter with CTCF from interacting with another CTCF site. While clusters 4 to 6, presented a loop pattern and were mostly associated to A compartment, were enriched in polycomb at both loop anchors, suggesting the formation of chromatin loops for a proper cell identity regulation. Together these findings define subcategories of CTCF–CTCF interactions within which only ∼30% (clusters 1 to 5) are consistent with the most accepted model of CTCF loops bringing together promoters and enhancers to initiate transcription (14). Additionally, the SOFM clustering allowed us to distinguish intermediate clusters, like cluster 6, that present some level of interaction between CTCF pairs, and thus could be interpreted as enhancer-promoter loops functionally related to polycomb.

The deconvolution of the average signal between CTCF–CTCF pairs allowed us thus to identify two major groups, one forming a canonical loop (clusters 1 to 5) and a second segregating CTCF sites (clusters 7 to 10). This allowed us to generate a *bona fide* set of CTCF–CTCF submatrices that generate loops versus those that do not. Next, we used those two sets to train an CNN to develop the loop-caller LOOPbit, which was technically and biologically tested against several previously benchmarked methods (23). LOOPbit, which was applied to 33 Hi-C experiments at 5 kb resolution and five experiments at 40 kb resolution, resulted in a number and length of loops similar to all other benchmarked methods and suffered from the same limitations. Indeed, loop-callers cannot easily reproduce findings when comparing replicates of low sequencing depths or binned at high resolutions. Fortunately, when high sequencing depth data is available, LOOPbit increased its reproducibility. Importantly, loops called by LOOPbit were found to be particularly relevant in terms of biological function. We found a clear enrichment of promoter-enhancer loops and depletion of loops between anchor points in heterochromatin state (9,14). When compared to other callers, LOOPbit detected almost twice as many promoter-enhancer loops. This feature is likely to be a direct consequence of the SOFM classification that allowed us to exclude from the training set chromatin loops mostly enriched in Polycomb or other repressed regions. Indeed, the SOFM classification was able to discern structural differences between repressed and promoter-enhancer loops, which then was learned by LOOPbit. Compared to other loop-callers, our methodology, prototyped in LOOPbit, is based on stratifying all possible structural pattern involving CTCF pairs, and selecting as a training set those matching the most the targeted promoter-enhancer loops. Most recent loop-callers as Peakachu (47) and Mustache (48) do not use this information. Peakachu, the most similar to LOOPbit, trains its RandomForest against CTCF peaks or H3K27ac marks but does not classify these pairs of coordinates to subset the most functionally relevant interaction patterns. LOOPbit finds CTCF loops enriched in active promoter–enhancer contacts as a consequence of being trained with such loops selected by our SOFM-UMAP classification. Interestingly, the loop-caller software that similarly performs to LOOPbit is HiCCUPS, a tool that uses a parallel approach: it searches for a specific interaction shape with local enrichment surrounded by less interactions with a major difference with LOOPbit being its specific training with active promoter-enhancer CTCF–CTCF loops.

In summary, we have shown that signal deconvolution is able to define sub-classes of CTCF-driven chromatin loops with specific structural features. We then designed a software based on this classification and its loops were the most enriched in enhancer–promoter interactions among other loops called by other software. With this work we have characterized and validated the relationship between structure and function of CTCF driven chromatin loops and provide a method that can be trained to identify chromatin loops driven by other DNA binding proteins beyond CTCF.

## DATA AVAILABILITY

Meta-Waffle as well as LOOPbit are available on GitHub: (https://github.com/3DGenomes/metawaffle and https://github.com/3DGenomes/loopbit, respectively).

## Supplementary Material

lqac021_Supplemental_File

## Notes

Present address: François Serra, Computational Biology Group - Barcelona Supercomputing Center (BSC), C/ Jordi Girona 29, 08034 Barcelona, Spain.
