# Supplementary material for: Identification of chromatin loops from Hi-C interaction matrices by CTCF–CTCF topology classification
Source: NAR Genom Bioinform. 2022 Mar 8;4(1):lqac021. doi: 10.1093/nargab/lqac021 (PMC8903010; doi:10.1093/nargab/lqac021)
Supplement: lqac021_Supplemental_File [file lqac021_supplemental_file.docx]

Supplementary Material to:

**Identification of chromatin loops from Hi-C interaction matrices by CTCF-CTCF topology classification**

Silvia Galan^1^, François Serra^1,#,^* and Marc A. Marti-Renom^1,^^2,3,4,^*

1. CNAG-CRG, Centre for Genomic Regulation (CRG), Barcelona Institute of Science and Technology (BIST), Baldiri i Reixac 4, 08028 Barcelona, Spain.

2. Centre for Genomic Regulation (CRG), Barcelona Institute of Science and Technology (BIST), Dr. Aiguader 88, 08003 Barcelona, Spain.

3. Universitat Pompeu Fabra (UPF), 08002 Barcelona, Spain.

4. ICREA, Pg. Lluís Companys 23, 08010 Barcelona, Spain.

#Current address: Computational Biology Group - Barcelona Supercomputing Center (BSC), C/ Jordi Girona 29, 08034, Barcelona, Spain

*To whom correspondence should be addressed: M.A.M-R. [martirenom@cnag.crg.eu](mailto:martirenom@cnag.crg.eu), F.S. [francois.serra@bsc.es](mailto:francois.serra@bsc.es)

**Supplementary Table 1.** GM12878 Hi-C experiments used to deconvolve the CTCF-CTCF topology (9).

| **ID** | **Reestriction enzyme** | **Filtered reads** |
| --- | --- | --- |
| GSM1551552 | MboI | 361,207,349 |
| GSM1551553 | MboI | 136,507,541 |
| GSM1551554 | MboI | 255,191,180 |
| GSM1551555 | MboI | 129,354491 |
| GSM1551556 | MboI | 148,151,521 |
| GSM1551557 | MboI | 172,219,884 |
| GSM1551558 | MboI | 99,044,057 |
| GSM1551559 | MboI | 48,503,486 |
| GSM1551560 | MboI | 48,052,662 |
| GSM1551561 | MboI | 115,277,508 |
| GSM1551562 | MboI | 58,351,032 |
| GSM1551563 | MboI | 209,244,601 |
| GSM1551564 | MboI | 94,795,755 |
| GSM1551565 | MboI | 94,272,224 |
| GSM1551566 | MboI | 147,025,950 |
| GSM1551567 | MboI | 119,160,683 |
| GSM1551571 | MboI | 228,444,864 |
| GSM1551572 | MboI | 218,414,758 |
| GSM1551573 | MboI | 77,453,007 |
| GSM1551577 | MboI | 54,523,851 |
| GSM1551578 | MboI | 121,751,924 |
| GSM1551588 | DpnII | 58,470,721 |
| GSM1551589 | DpnII | 76,770,682 |
| GSM1551590 | DpnII | 61,529,714 |
| GSM1551591 | DpnII | 93,923,134 |
| GSM1551598 | MboI | 82,406,062 |

**Supplementary Table 2.** Hi-C experiments used for LOOPbit benchmarking. All the experiments were pre-processed and filtered using TADbit (28) and OneD normalized (29).

| **Dataset** | **ID** | **Restriction enzyme** | **Cell type** | **Filtered reads** | **Resolution**  **(kb)** |
| --- | --- | --- | --- | --- | --- |
| JIN | GSM1055805 | HindIII | H1-hESC | 134,678,276 | 5 |
| JIN | GSM1055800 | HindIII | IMR90 | 104,929,328 | 5 |
| JIN | GSM1055801 | HindIII | IMR90 | 175,071,658 | 5 |
| JIN | GSM1154021 | HindIII | IMR90 | 97,395,328 | 5 |
| JIN | GSM1154022 | HindIII | IMR90 | 79,559,232 | 5 |
| JIN | GSM1154023 | HindIII | IMR90 | 52,425,794 | 5 |
| JIN | GSM1154024 | HindIII | IMR90 | 54,082,516 | 5 |
| RAO | GSM1551552 | MboI | GM12878 | 361,207,349 | 5 |
| RAO | GSM1551569 | MboI | GM12878 | 72,934,660 | 5 |
| RAO | GSM1551570 | MboI | GM12878 | 77,651,974 | 5 |
| RAO | GSM1551571 | MboI | GM12878 | 228,444,864 | 5 |
| RAO | GSM1551572 | MboI | GM12878 | 218,414,758 | 5 |
| RAO | GSM1551573 | MboI | GM12878 | 77,453,007 | 5 |
| RAO | GSM1551574 | MboI | GM12878 | 81,318,602 | 5 |
| RAO | GSM1551575 | MboI | GM12878 | 80,613,339 | 5 |
| RAO | GSM1551576 | MboI | GM12878 | 80,438,152 | 5 |
| RAO | GSM1551577 | MboI | GM12878 | 54,523,851 | 5 |
| RAO | GSM1551578 | MboI | GM12878 | 121,751,924 | 5 |
| RAO | GSM1551587 | DpnII | GM12878 | 63,854,975 | 5 |
| RAO | GSM1551588 | DpnII | GM12878 | 58,470,721 | 5 |
| RAO | GSM1551589 | DpnII | GM12878 | 76,770,682 | 5 |
| RAO | GSM1551590 | DpnII | GM12878 | 61,529,714 | 5 |
| RAO | GSM1551591 | DpnII | GM12878 | 93,923,134 | 5 |
| rao | GSM1551599 | MboI | IMR90 | 164,365,813 | 5 |
| rao | GSM1551600 | MboI | IMR90 | 181,640,359 | 5 |
| rao | GSM1551601 | MboI | IMR90 | 20,676,015 | 5 |
| rao | GSM1551602 | MboI | IMR90 | 90,970,187 | 5 |
| rao | GSM1551603 | MboI | IMR90 | 186,707,523 | 5 |
| rao | GSM1551604 | MboI | IMR90 | 198,492,270 | 5 |
| rao | GSM1551605 | MboI | IMR90 | 216,948,852 | 5 |
| Dixon 2015 | GSM1267196 | HindIII | H1-hESC | 172,971,685 | 5 |
| dixon 2015 | GSM1267197 | HindIII | H1-hESC | 103,476,074 | 5 |
| Sexton | GSM849422 | DpnII | Fly embryo | 31,357,023 | 40 |
| dixon 2012 | GSM862723 | HindIII | H1-hESC | 21,292,727 | 40 |
| dixon 2012 | GSM892306 | HindIII | H1-hESC | 134,678,276 | 40 |
| dixon 2012 | GSM862724 | HindIII | IMR90 | 102,906,483 | 40 |
| dixon 2012 | GSM892307 | HindIII | IMR90 | 104,974,904 | 40 |

**Supplementary Table 3.** CTCF ChIP-seq experiments used in the analysis.

| **Experiment** | **Cell type** | **Accession number** |
| --- | --- | --- |
| ctcf cHip-SEQ | H1-hESC, GM12878 | GSE29611 |
| ctcf cHip-SEQ | IMR90 | GSE31477 |
| ctcf cHip-SEQ | Embryo 14-16hr Oregon-R | GSE47264 |

**Supplementary Figure 1. SOFM examples.** Examples of SOFM results with different combination of parameters (grid size: GS; learning radius: LR; standard deviation: STD; step size: STEP; number of epochs: EPOCH). Each cell shows the median interaction pattern between CTCF-CTCF pairs (red: high interaction count; blue: low interaction count).


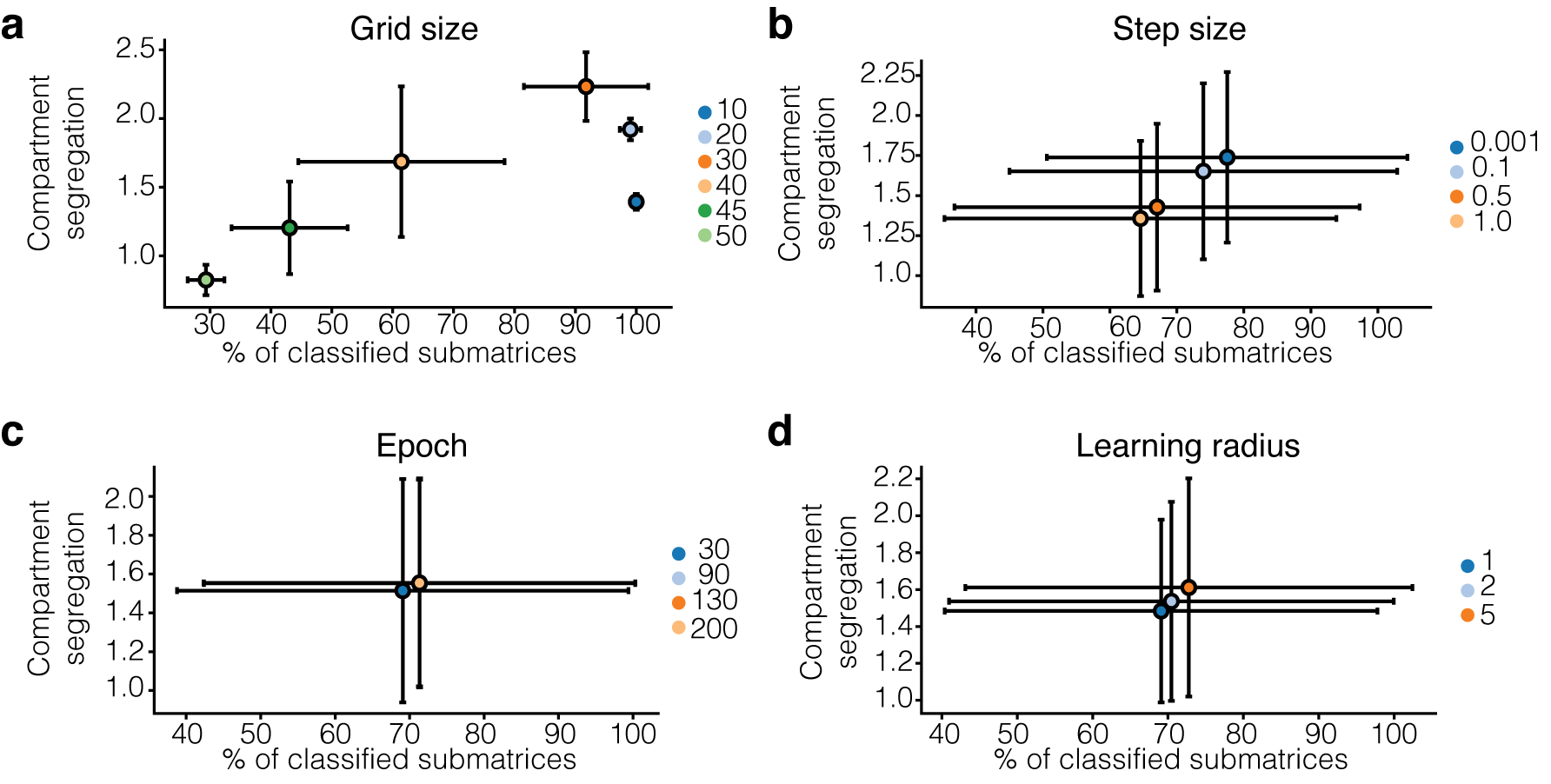


**Supplementary Figure 2. SOFM parameters.** SOFM parameters, grid size, step size, epoch and learning radius, based on the percentage of classified matrices and the compartment segregation value (see also **Fig. 1**), **a**, **b**, **c** and **d,** respectively.

**Supplementary Figure 3.** **Enrichment in RAD21(A) and SMC3 (B)** along each of the 10 clusters of CTCF-CTCF pairs. RAD21 and SMC3 here downloaded from ENCODE (https://www.encodeproject.org) with the respective accession numbers ENCSR000BMY and ENCSR000DZP.


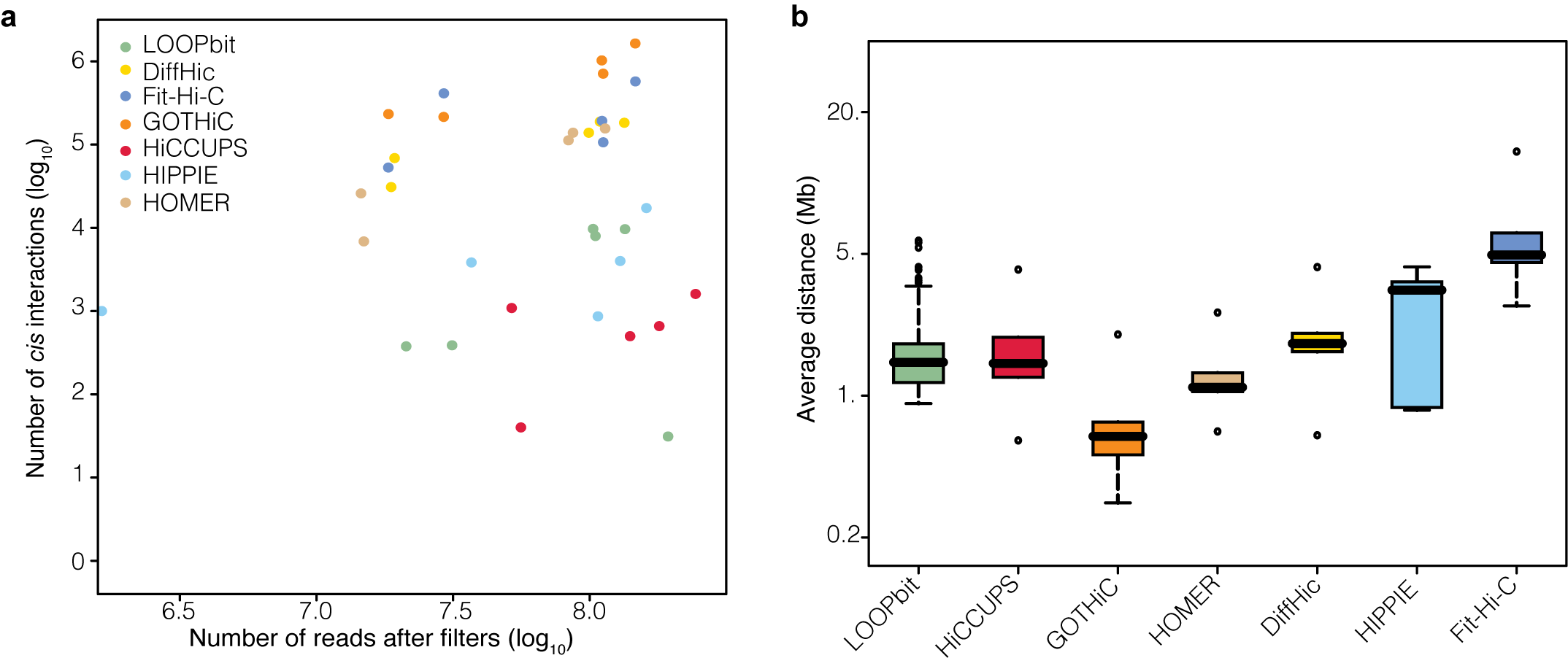


**Supplementary Figure 4. Results benchmark at 40 kb resolution. a.** Number of reads after filters and the number of identified cis-interactions by LOOPbit in all experiments at a 40 kb resolution (n=5). **b.** Average distance between the identified loop-anchors of all the Hi-C experiments at 40 kb resolution (n=5).


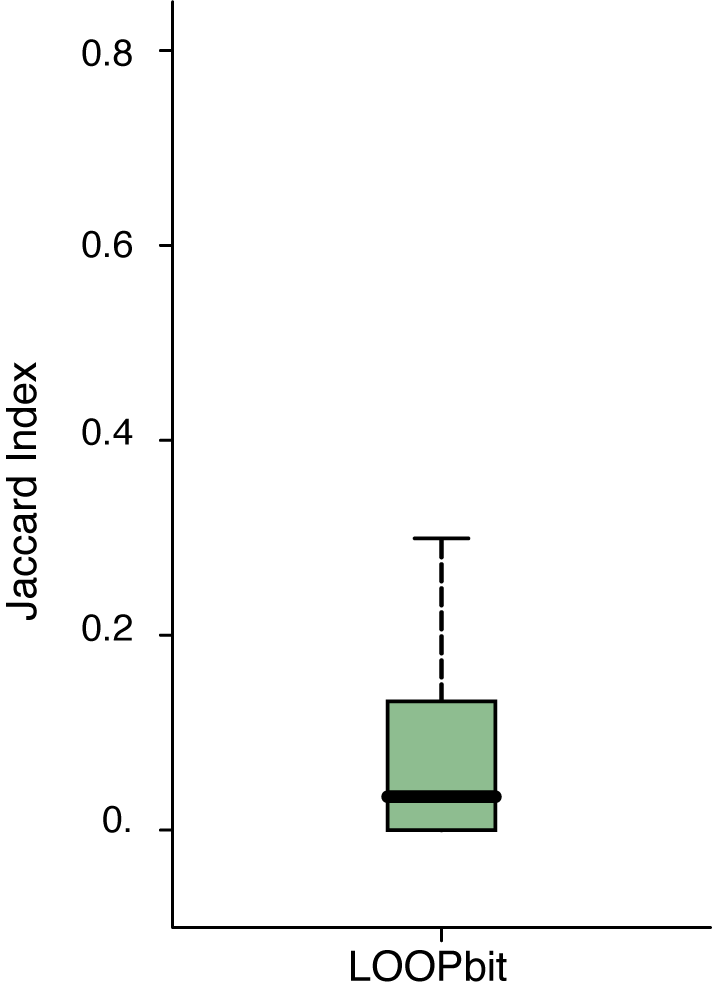


**Supplementary Figure 5. Jaccard Index allowing 70% of overlapping between chromatin loops.** Boxplot representing the Jaccard Index, in here an overlap of 70% between chromatin loops were considered to be the same loop between the same replicates (n=39).


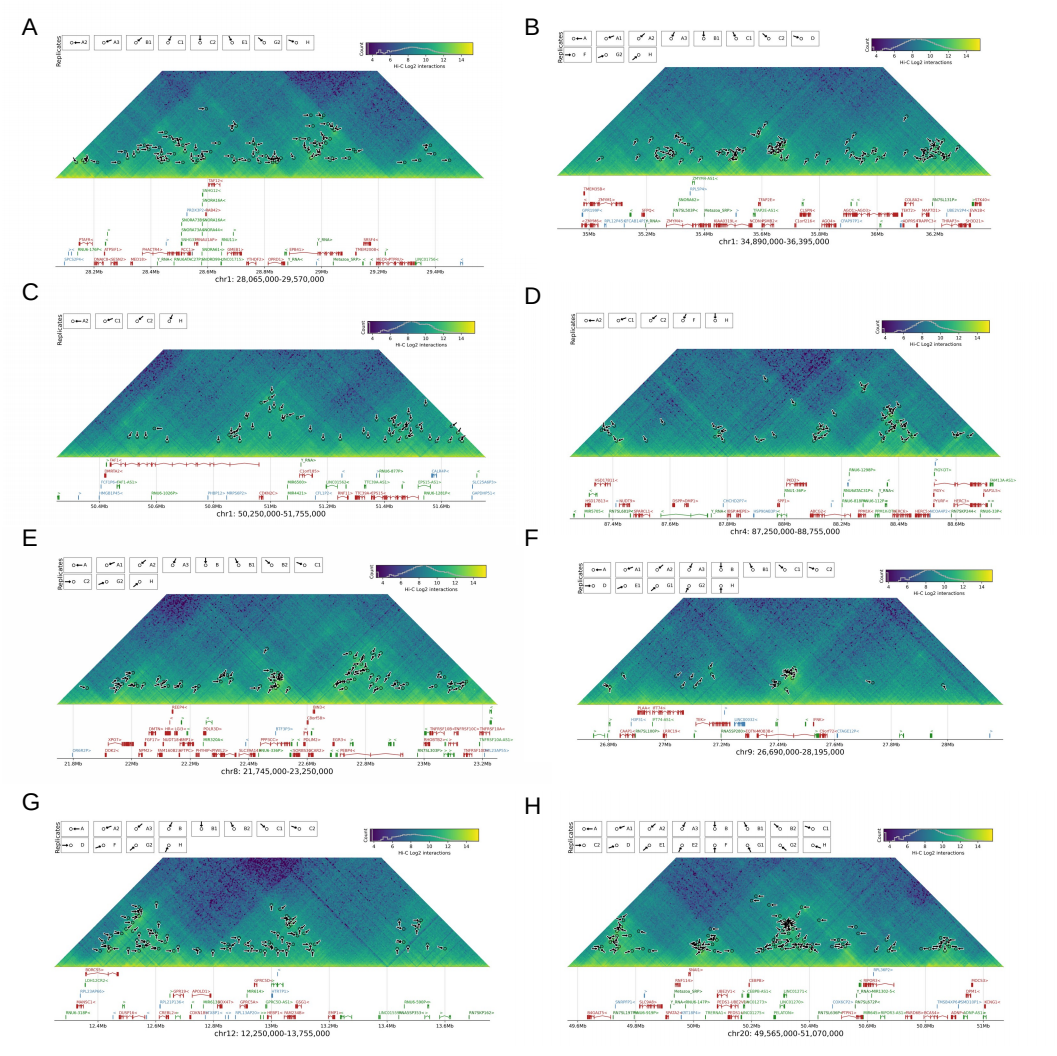
**Supplementary Figure 6. Examples of LOOPbit results in several GM12878 replicates** (5 kb resolution)**.** Result of loop calling in a chunk of human chromosome 22. Arrows indicate predicted loops in different sub-samples of GM12878 (different arrow orientations correspond to different sub-samples). The Hi-C matrix represents the sum of interactions from all GM12878 replicates available.


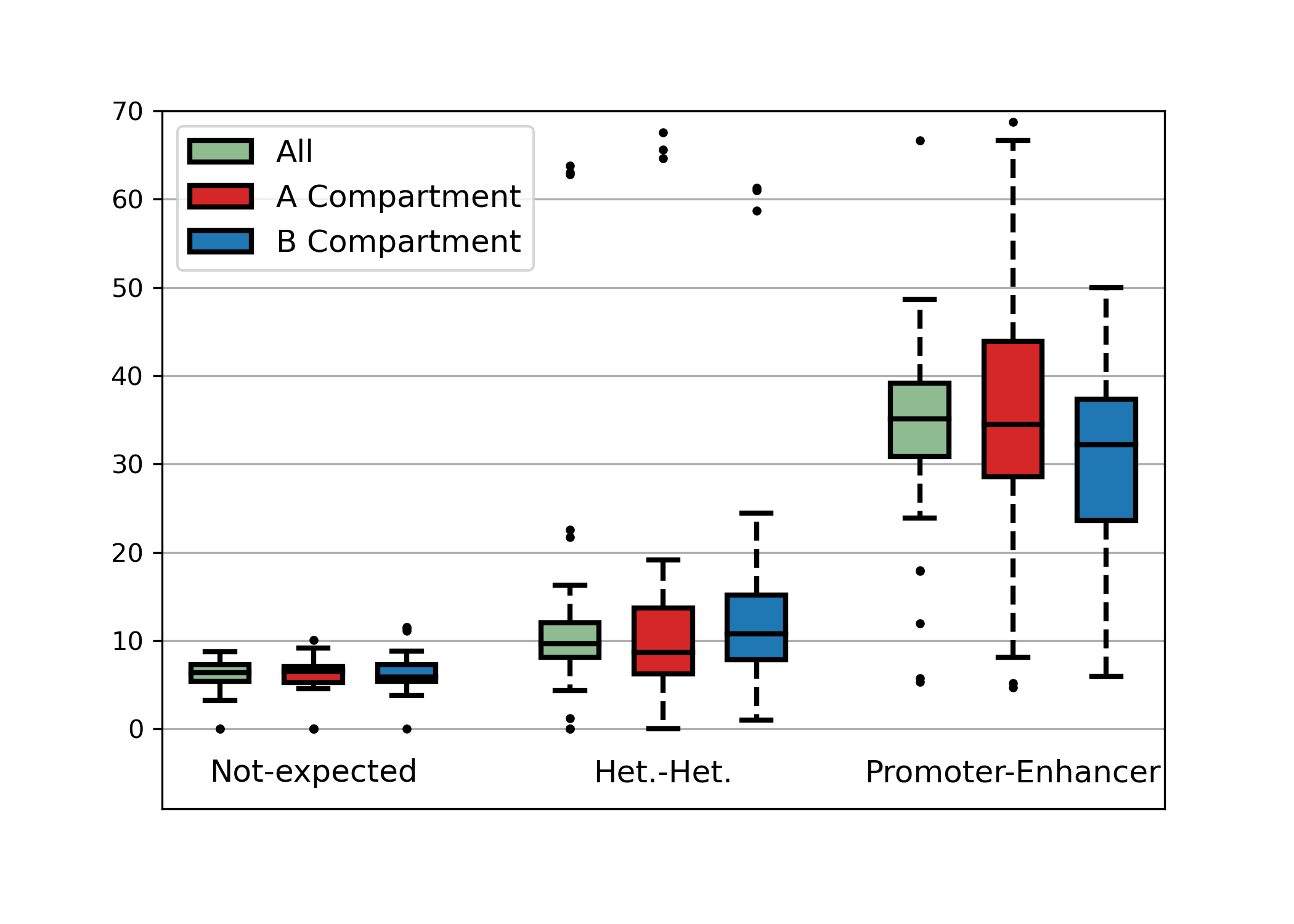
**Supplementary Figure 7: CTCF-CTCF loop enrichment in chromatin states per compartment type.** Same as Figure 3e, but showing loops with both anchor in A-type compartments (red) or both anchors in B-type compartments (blue).
